# Quality of Life and Fatigue in Inflammatory Bowel Disease: A Systematic Review

**DOI:** 10.3390/healthcare13172203

**Published:** 2025-09-03

**Authors:** Maheeba Abdulla, Nafeesa Mohammed, Jehad AlQamish, Bisher Sawaf

**Affiliations:** 1 Department of Internal Medicine, Ibn Al Nafees Hospital, Road 3302, Manama P.O. Box 54533, Bahrain; 2 Internal Medicine Department, Salmaniya Medical Complex, Manama 317, Bahrain; 3 Department of Internal Medicine, The University of Toledo Medical Center, Toledo, OH 43614, USA

**Keywords:** inflammatory bowel disease, quality of life, fatigue, healthy controls, IBD

## Abstract

**Objective**: The objective of this paper is to assess the quality of life and fatigue among adult patients diagnosed with inflammatory bowel disease (IBD) compared to healthy controls. **Methods**: We conducted searches in Medline and Embase from January 2015 to April 2025. We included publications that examined adult patients with IBD without comorbidities, compared them to healthy controls, and assessed quality of life and fatigue. Two reviewers independently conducted study selection, data extraction, and quality assessment. We described results narratively due to high heterogeneity. **Results**: Ten studies with a pool of 10,661 participants were selected. Three studies showed significantly worsened mental and physical components for quality of life in IBD patients compared to healthy controls. Additionally, three studies reported that fatigue was more prevalent among patients with IBD than in healthy controls. **Conclusions**: Our review suggests that quality of life is significantly reduced, and fatigue is more prevalent in IBD patients compared to healthy controls. Further long-term follow-up studies are needed to provide a more comprehensive understanding of the global impact of IBD, particularly in non-Western populations.

## 1. Introduction

Patients’ health-related quality of life (HRQoL) is significantly impacted by inflammatory bowel diseases (IBDs) [1]. Fatigue, rectal blood loss, and abdominal pain are all primary symptoms of IBD. Even among patients with quiescent disease, 40% report experiencing fatigue [2]. IBD patients may endure embarrassing and painful symptoms, a chronic and unpredictable disease course, and anxiety about various life-related complications, including fatigue, social isolation, bowel control, and concerns about developing cancer or requiring surgery [3]. Prior to recent advancements in clinical trial designs and regulations emphasizing patient-reported outcomes as primary endpoints, HRQoL and related psychosocial measures were rarely investigated in clinical trials. However, these measures are now gaining importance [4].

Individuals with chronic illnesses such as IBD report experiencing fatigue in a distinct and more intense manner than healthy individuals [5]. Most prevalence studies on IBD have originated from Western countries [6], and in-depth analyses of the disease burden in Asia remain in their early stages. The physiological pathways and mechanism of fatigue in IBD remain poorly understood [7].

This fatigue is typically at least two times more severe than healthy controls and has a detrimental impact on QoL [8,9]. Despite this, research on fatigue in IBD remains limited [10].

Additionally, research on QoL and fatigue in IBD patients with varying levels of disease activity remains scarce, with several controversial aspects still unresolved. Hence, in this study, we assess health-related QoL and fatigue in IBD patients.

## 2. Materials and Methods

### 2.1. Objective

This systematic review aims to determine the quality of life (QoL) and fatigue among adult patients with inflammatory bowel disease (IBD) compared to healthy controls.

This systematic review was conducted and reported following the PRISMA statement guidelines [11]. This study was registered with PROSPERO, registration number CRD420251072983.

### 2.2. Search Strategy

We developed search strategies by combining terms related to inflammatory bowel disease and quality of life. A well-conducted systematic review was conducted, and searches were updated until 2015; hence, we used that year as a cut-off to conduct our searches [12]. Searches were conducted in both Medline and Embase databases, limited to the English language and the period from 1 January 2015 to 15 April 2025. Full search strategies are reported in Supplementary Materials. The retrieved search results were exported into Endnote for duplicate removal.

### 2.3. Eligibility Criteria

To be included, studies met the following criteria:

#### 2.3.1. Inclusion Criteria

- Studies must have investigated IBD (including ulcerative colitis and Crohn’s disease) with established diagnostic parameters;
- Studies must have graded quality of life using any validated instrument;
- Studies must have assessed fatigue using any validated instrument;
- Studies must involve an adult study population;
- Studies must have compared quality of life in people diagnosed with IBD with a normal population;
- Studies must have designed and included randomized controlled trials, cross-sectional, retrospective, prospective, and other controlled studies.

#### 2.3.2. Exclusion Criteria

- Studies investigating non-fatigue comorbidities;
- Conference abstracts, posters, commentaries, and letters;
- Case reports, case series, or qualitative research;
- Animal studies.

### 2.4. Study Selection

Two independent reviewers screened the abstract and title for all selected articles. Disagreements on screening and selection were solved by considering opinions from a third independent reviewer. Using the predefined selection criteria, full-text articles were retrieved for all selected studies. Disagreements at this stage were also solved with the expert opinion of a third independent reviewer. The Rayyan tool was used for the study selection process [13].

### 2.5. Data Extraction

Data about the following variables were extracted:

- Authors, publication year, study design, and setting;
- Participant characteristics, including IBD subtype, sex, disease activity status, and sample size;
- Outcome measures and results for the primary outcomes.

Two independent reviewers conducted data extraction and documentation using a standardized data collection form.

### 2.6. Quality Assessment Studies

The quality of all selected studies was assessed by two reviewers. Disagreements were resolved by considering the expert opinion of a third independent reviewer. Cohort studies were appraised using the Newcastle–Ottawa Scale (NOS) [14]. Cross-sectional studies were evaluated using an adapted NOS.

### 2.7. Data Synthesis

A narrative synthesis was provided for all included studies. We planned to conduct random-effects meta-analyses with the DerSimonian and Laird method [15]; however, they were not conducted because of heterogeneity.

## 3. Results

In total, 5486 studies were considered through database searches. After excluding duplicate studies (n = 2433), 3053 unique articles were screened and selected based on titles and abstracts. Post-screening assessment excluded another 3013 articles irrelevant to the objective of this study.

- 40 studies underwent full-text assessment;
- Of all 40, only 10 studies were included in the final analysis for meeting the inclusion parameters.

### 3.1. Study Characteristics

**Figure 1:**
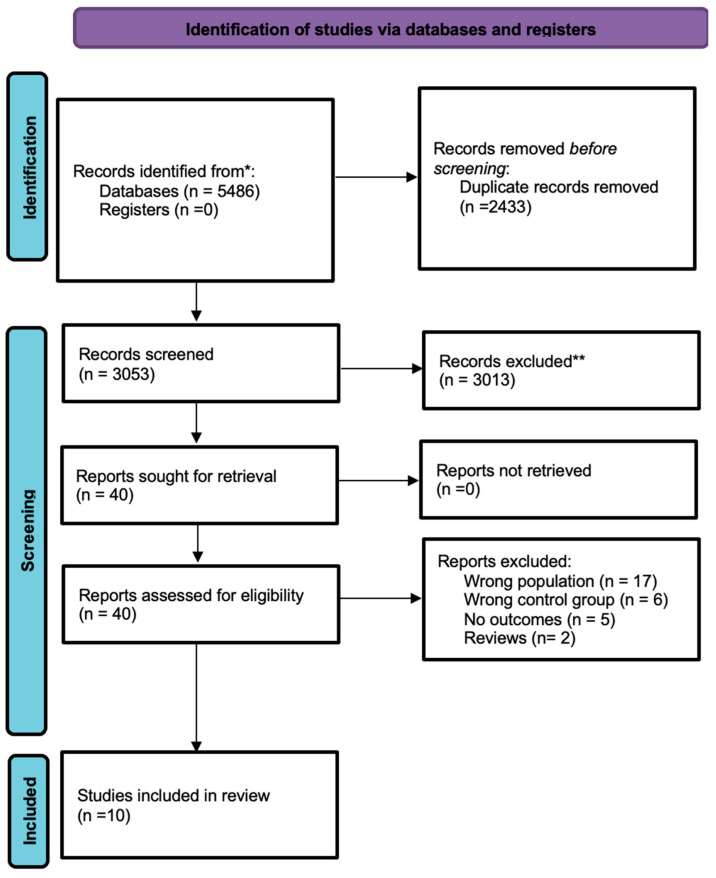
Study selection flow. *: all mentioned databases; **: after title and abstracts screening.

Figure 1 represents flow of study selection and characteristics of included studies reported in Table 1.

- A total of 10,661 participants were included.
- Study designs:
  ⚬ Cross-sectional studies (n = 4);
  ⚬ Cohort studies (n = 6).
- Study settings:
  ⚬ Hospital-based studies (n = 6);
  ⚬ Community-based studies (n = 3).
- Median follow-up duration: 4.89 years (range: 0.12–8.66 years);
- Gender distribution: 54% male, 46% female;
- The quality of every study included was graded from good to fair quality (Tables S1 and S2);
- Cross-sectional studies included fewer than 400 participants, while all other studies received high scores for selection, comparability, and outcome assessment.

### 3.2. Quality of Life

Six studies assessed QoL among IBD participants compared to healthy controls [16,17,18,21,22,23]. Of these, four studies employed the SF-36 questionnaire, one study used SF-12, and another used QLQ-CR29 [21]. Three studies that used SF-36 and assessed both the mental and physical components of QoL, which reported significantly lower scores for IBD patients than control groups. Other studies reported that QoL was significantly impacted in IBD participants. Another study used the Portuguese version of the Short Inflammatory Bowel Disease Questionnaire (SIBDQ) for measuring the HRQoL [24]. Among the remaining two studies, one study used the SF-12 questionnaire, which indicated that compared to healthy controls, QoL’s physical and mental components were significantly lower in IBD patients. Another study used QLQ-CR29, which also demonstrated poor QoL in IBD patients compared to healthy controls. Comparison of mean SIBDQ scores were higher in patients with non-CD than with CD (56.8 ± 9.6 vs. 53.2 ± 32.0; *p* = 0.034) [24].

### 3.3. Fatigue

Only three studies reported data on fatigue outcomes [5,19,20]; however, due to heterogeneity, we did not conduct a meta-analysis. Of these three studies, two used the Fatigue Severity Scale and one study used the Generic Fatigue Questionnaire. Schreiner et al.’s 2021 study [5] found that more than half of the IBD patients (672/1208) reported significant fatigue compared to healthy controls (145/414) (OR 2.71; 95% CI 2.08–3.54; *p* < 0.001). In addition, IBD also affected daily activities. Holten et al.’s 2023 [20] study also found that two-thirds of patients newly diagnosed with IBD had experienced fatigue. In addition, Grimstad et al.’s 2021 [19] study reported that IBD patients who had undergone conventional treatment also reported fatigue more than healthy controls.

## 4. Discussion

Our systematic review indicates a primary finding: compared to healthy populations, the physical and mental components of life quality are significantly lower in IBD patients. Both components were found to be lower in all adult IBD patients. Additionally, fatigue was also significantly more prevalent in IBD patients.

Our findings align with previous research findings [12]. In both interventional and observational studies, QoL is considered a significant indicator of clinical outcomes in the management of IBD [12]. This study primarily provides a broad comparison of QoL between adult IBD patients and healthy controls while also investigating the impact of IBD on fatigue prevalence and HRQoL.

Our findings support previous research demonstrating the extensive and multifaceted impact of IBD on individuals’ lives. Research consistently shows that IBD significantly lowers HRQoL across social, psychological, and physical domains. According to Lonnfors et al. [1], IBD patients frequently experience persistent symptoms such as diarrhea, rectal bleeding, and abdominal pain, which negatively impact their day-to-day activities and social relationships.

These symptoms further reduce QoL by leading to frequent hospital visits and prolonged hospital stays. Romberg-Camps et al. [25] showed a strong correlation between psychological distress and lower HRQoL, indicating a high prevalence of depression and anxiety in IBD. Moreover, several studies, including Minderhoud et al. [8], have shown that fatigue is a common and severe symptom of IBD, affecting nearly 40% of patients even during remission.

Our results align with those of Chavarría et al. [26], who found that fatigue significantly lowers QoL, with a high severity in IBD patients compared to healthy individuals. This observation suggests that fatigue is not merely an outcome of an active disease state but rather a core component of the disease burden.

IBD fatigue is multifactorial, involving both psychological and physiological factors. According to Borren et al. [7], fatigue may persist even in quiescent disease due to underlying mechanisms such as chronic low-grade inflammation. The fact that fatigue is multifaceted emphasizes the need for a comprehensive management strategy.

A holistic strategy that incorporates lifestyle changes, such as regular exercise and stress-reduction methods, has been shown to be beneficial, as noted by Artom et al. [27]. For IBD patients, taking care of these issues can greatly enhance their QoL.

Our findings show that most studies on IBD and its effects were conducted in Western countries. Burisch et al. [28] and Zhao et al. [29] have highlighted a significant research gap in understanding the disease burden and patient experiences in non-Western populations. Expanding research to diverse geographic and cultural contexts significantly improves the global understanding of IBD, bridging the gap between research and clinical practice.

A few limitations in our findings should be acknowledged. In terms of study design, we selected three cross-sectional studies, limiting the ability to determine causal relationships. Regarding the heterogeneity in measurement tools, different studies used varied QoL and fatigue measurement instruments, making direct comparisons challenging. In addition, we did not conduct a reporting bias assessment due to no meta-analysis being conducted.

Future research should prioritize longitudinal studies, as they better show correlations among fatigue, QoL, and disease progression. Additionally, including more multinational studies may better explore the global symptomatic burden of IBD across diverse populations.

## 5. Conclusions

Our systematic review demonstrates that clinical indications of a worsened quality of life (QoL) are significantly prominent in IBD patients compared to healthy individuals, affecting both mental and physical health domains. Even in clinical remission, fatigue—a prevalent symptom in IBD—persists. This observation suggests that fatigue is not solely a consequence of active disease but a core element of the disease burden, requiring better recognition and management.

Given the rising global incidence of IBD, future research should focus on longitudinal, multinational studies to explore sociocultural variations, disease burden, and patient experiences in non-Western populations. Standardizing QoL and fatigue measurement tools will improve comparability and facilitate meta-analyses across studies.

To optimize patient care, healthcare providers should consider a holistic, patient-centric approach, ensuring that both physical and psychological health are addressed. Further global research is needed to bridge knowledge gaps and develop comprehensive, multidisciplinary management strategies for IBD patients worldwide.

## Figures and Tables

**Table 1:** Study characteristics.

SN	Study Author, Year	Study Design	Setting	Study Duration	Participant Characteristics (Sample Size)	% of Gender	Measures of QoL	Measures of Fatigue
1	AlQahtani, 2022 [16]	Cross-sectional	Community	45 days	Crohn’s disease: 109 Healthy control: 370	Crohn’s disease M: 55% F: 45% Control M: 26% F: 74%	SF-36	NR
2	Bulut, 2019 [17]	Cross-sectional	Community	9 months	IBD:122 Control:42	CD: M: 41.3% F: 58.6% UC: M: 54.68% F: 45.31% Control M: 47.6% F: 52.38%	SF-36	NR
3	Bogut, 2022 [18]	Cross-sectional	Hospital	6 months	IBS: 40 IBD: 40 Control: 40	(IBS): M: 0% F:40% IBD: M: 55%; F: 45% Control: M: 42.5%; F: 57.5%	SF-36	NR
4	Grimstad, 2022 [19]	Cohort	Hospital	5 years, 9 months	UC: 149 Control: 22	UC: M: 54.3%; F: 45.7% Control M: 45.5%; F: 54.5%	NR	Fatigue Severity Scale (FSS)
5	Holten, 2023 [20]	Cohort	Hospital	3 years	IBD: 983 Control: 2287	UC vs. Control M: 49.6%; F: 72.1% CD vs. Control M: 56.2%; F: 79.2%	NR	Generic Fatigue Questionnaire [FQ]
6	Kunovsky, 2018 [21]	Cohort	Hospital	6 years	CD: 215 Control: 104	Operated: M: 44.7%; F: 55.3% Non-operated: M: 33.7%; F: 66.3%; Control: M: 40.4%; F: 59.6%	QLQ-CR29 questionnaire	NR
7	Ling, 2021 [22]	Cohort	Hospital	2 years	Diseased: 112 Control: 165	IBD: M: 67.9%; F: 32.1% UC: M: 66.7%; F: 33.3% CD: M: 68.5%; F: 31.5% Control: M: 67.9%; F: 32.1%	SF-36	NR
8	Schreiner, 2021 [5]	Cohort	Hospital	1.2 years	IBD: 1208 Control: 414	IBD: M: 63.4%; Control M: 41.2%	NR	Visual analogue scale and Fatigue Severity Scale (FSS)
9	Trieschmann, 2022 [23]	Cohort	Community advertisement	8 years, 8 months	IBD: 74 IBS: 74 Control: 74	Diseased IBD: F: 40% IBS: F: 40% Control: F: 40%	SF-12	NR
10	Oliveira, 2024 [24]	Cross-sectional case–control study	Hospital	NA	CD: 69 UC: 170 Control: 126	CD: 63.8 UC: 53.5 Control: 46.8	Short Inflammatory Bowel Disease Questionnaire	NR

CD: Crohn’s disease; IBD: inflammatory bowel disease; NA: not applicable; UC: ulcerative colitis; IBS: inflammatory bowel syndrome; M: male; F: female; NR: not reported.

## Data Availability

All the data and materials related to this article are available in this manuscript. Any additional data can be made available from the corresponding author on appropriate request.

## Supplementary Materials

The following supporting information can be downloaded at: https://www.mdpi.com/article/10.3390/healthcare13172203/s1, Table S1: Newcastle–Ottawa Scale quality assessment of the included studies (details). Cohort Star Template; Table S2: Newcastle–Ottawa Scale quality assessment of the included studies (details). Cross-sectional studies Star Template.

## Abbreviations

The following abbreviations are used in this manuscript:

HRQoL	Multidisciplinary Digital Publishing Institute
IBD	inflammatory bowel diseases
QoL	quality of life
MDs	mean differences
SIBDQ	Short Inflammatory Bowel Disease Questionnaire
CD	Chron’s disease
