# Supplementary material for: Quality of Life and Fatigue in Inflammatory Bowel Disease: A Systematic Review
Source: Healthcare (Basel). 2025 Sep 3;13(17):2203. doi: 10.3390/healthcare13172203 (PMC12427836; doi:10.3390/healthcare13172203)
Supplement: Supplementary file 1 [file healthcare-13-02203-s001.zip › healthcare-3661907-supplementary.pdf]

healthcare

MDPI

Supplementary Materials

Search strategy: Medline and Embase

1 exp Inflammatory Bowel Diseases/

2 (Inflammatory bowel disease* or IBD).tw,kw.

3 (Crohn* or ileitis or regional enteritis or ileocolitis or granulomatous colitis orgranulomatous enteritis).tw,kw.

4 (colitis or proctocolitis or proctosigmoiditis or proctitis or rectosigmoiditis or rectocolitis orcolorectitis or coloproctitis).tw,kw.

5 or/1-4

6 "Quality of Life"/

7 (qualit* adj2 life).tw.

8 Fatigue/

9 or/6-8

10 5 and 9

11 limit 10 to (english language and humans)

Table S1. Newcastle–Ottawa Scale quality assessment of the included studies (details). Cohort Star Template.

Study[1]	Selection of cohorts				Comparability of cohorts	Outcome			Power[2]
	Representativeness of the exposed cohort	Selection of the non exposed cohort	Ascertainment of exposure	Demonstration that outcome of interest was not present at start of study	Comparability of cohorts on the basis of the design or analysis	Assessment of outcome	Was follow up long enough for outcomes to occur	Adequacy of follow up of cohorts	
Grimstad, 2021	☆	☆	☆	☆	☆☆	☆	☆	☆	Good Quality
Holten, 2023	☆	☆	☆	☆	☆☆	☆	☆	☆	Good Quality
Kunovsky, 2019	☆	☆		☆	☆☆		☆		Poor Quality
Ling, 2021	☆	☆	☆	☆	☆☆	☆	☆	☆	Good quality
Schreiner, 2020	☆	☆	☆	☆	☆☆	☆	☆	☆	Good quality
Trieschmann, 2023	☆	☆	☆	☆	☆	☆	☆		Fair quality

Note.
[1] To reduce the risk of bias, each article was assessed by an independent judge, and no discrepancies were found between both reviewers
[2] Good quality: 3 or 4 stars in selection domain AND 1 or 2 stars in comparability domain AND 2 or 3 stars in outcome/exposure domain; Fair quality: 2 stars in selection domain AND 1 or 2 stars in comparability domain AND 2 or 3 stars in outcome/exposure domain; Poor quality: 0 or 1 star in selection domain OR 0 stars in comparability domain OR 0 or 1 stars in outcome/exposure domain
[3] Low risk of bias = *Good and fair quality* // High risk of bias: *Poor quality*

From: Wells, G.A.; Shea, B.; O'Connell, D.; Peterson, J.; Welch, V.; Losos, M.; Tugwell, P. (2000). The Newcastle-Ottawa Scale (NOS) for Assessing the Quality of Nonrandomised Studies in Meta-Analyses. 2000. Available online: https://www.ohri.ca/programs/clinical_epidemiology/oxford.asp Lorem Ipsum

Table S2. Newcastle–Ottawa Scale quality assessment of the included studies (details). Cross-sectional studies Star Template.

Study[1]	Selection of cohorts				Comparability of cohorts	Outcome			Power[2]
	Representativeness of the sample	Sample Size	Non-respondents	Ascertainment of exposure	Comparability of cohorts on the basis of the design or analysis	Assessment of outcome		Statistical test	
AlHarbi, 2022	☆	☆	☆	☆	☆☆	☆		☆	Good quality[3]
Bulut, 2019	☆	☆	☆	☆	☆	☆		☆	Poor Quality
Bogut, 2022	☆	☆	☆	☆	☆	☆		☆	Poor Quality
Oliveira, 2024	☆	☆		☆	☆☆ ☆☆	☆	☆	☆	Poor Quality Good quality[3]

Note.
[1] To reduce the risk of bias, each article was assessed by an independent judge, and no discrepancies were found between both reviewers
[2] Good quality: 3 or 4 stars in selection domain AND 1 or 2 stars in comparability domain AND 2 or 3 stars in outcome/exposure domain; Fair quality: 2 stars in selection domain AND 1 or 2 stars in comparability domain AND 2 or 3 stars in outcome/exposure domain; Poor quality: 0 or 1 star in selection domain OR 0 stars in comparability domain OR 0 or 1 stars in outcome/exposure domain
[3] Low risk of bias = *Good and fair quality* // High risk of bias: *Poor quality*

From: Wells, G.A.; Shea, B.; O'Connell, D.; Peterson, J.; Welch, V.; Losos, M.; Tugwell, P. (2000). The Newcastle-Ottawa Scale (NOS) for Assessing the Quality of Nonrandomised Studies in Meta-Analyses. 2000. Available online: https://www.ohri.ca/programs/clinical_epidemiology/oxford.asp Lorem Ipsum
